# Secondary Merkel Cell Carcinoma Manifested in the Parotid

**DOI:** 10.1155/2013/960140

**Published:** 2013-11-27

**Authors:** M. Basati, K. Kassam, A. Messiha

**Affiliations:** Northwick Park Hospital, Watford Road, Harrow HA1 3UJ, UK

## Abstract

*Background*. Merkel cell carcinoma of the head and neck is a rare and aggressive malignant tumour. Both the dermatological and surgical colleagues should be aware of this entity as lesions usually present on sun exposed areas of the skin such as the head and neck. *Main Observation and Treatment*. A 69-year-old male originally presented to the maxillofacial surgery department with a growing lesion on the left eyebrow. Histological analysis confirmed Merkel cell carcinoma and consequently surgical excision was carried out. A follow-up PET/CT scan 2 years later demonstrated a hotspot in the left parotid gland. Fine needle aspiration and cytology revealed Merkel cell carcinoma. A subtotal parotidectomy left side with ipsilateral selective neck dissection levels I to III was carried out. *Conclusions*. Potential secondary Merkel cell carcinoma in the head and neck region should be taken into account when planning short- and long-term follow up for previously diagnosed patients. This followup should involve both dermatological and surgical colleagues.

## 1. Introduction

Merkel cell carcinoma (MCC) is a rare and aggressive malignant tumour of neuroendocrine origin, with incidence being reported as low as 0.44/100 000 cases a year [1]. Clinical diagnosis is difficult due to a nonspecific appearance. Often the lesion presents as a nonindurated and slightly erythematous nodule. The most common location of the lesion is on sun exposed areas of the skin, with UVB radiation posing an increased risk [2]. Epidemiological studies reveal further risk in immunocompromised patients and Caucasian patients older than 50 [3]. Furthermore 48% of lesions are diagnosed in the head and neck region, with 61% of patients being male [4]. Heath et al. [3] use a favourable acronym when looking at typical clinical features at presentation: AEIOU, asymptomatic, enlarging rapidly, immunosuppression, older age, and UV exposed site.

MCC has a poor 5-year prognosis, with a 75% survival for local disease and 50% for regional [5]. The prognosis is even poorer for: (1) male patients, (2) primaries of T2 size and extension, (3) nodal involvement, (4) and if metastatic disease is present [4]. The frequency of both local and regional spread of disease is high, with up to 21% of cases developing distant metastatic lesions [6]. Common sites of metastasis have been described as lymph nodes, mediastinum, lung, liver, and bone [7]. The mortality rate of MCC is twice that of melanoma [8]. Even with this aggressive nature of the disease and high risk of mortality, the awareness of MCC is poor amongst practitioners. The following case report aims to increase awareness and stimulate debate and education within units.

## 2. Case Presentation

A 69-year-old male originally presented to the oral and maxillofacial department in 2010 with a gradually increasing lump in the left eyebrow region of four weeks duration. A diagnosis of localised MCC was made following wide local excision. There was total eradication of residual disease. The patient was under regular follow-up scans and in 2012 a PET/CT revealed a 1.3 cm left intraparotid node of uncertain significance.

Medical history to note was noninsulin dependent diabetes, hypertension, and atrial fibrillation. The patient had good social support, living with wife and extended family.

## 3. Investigations

**Figure 1:**
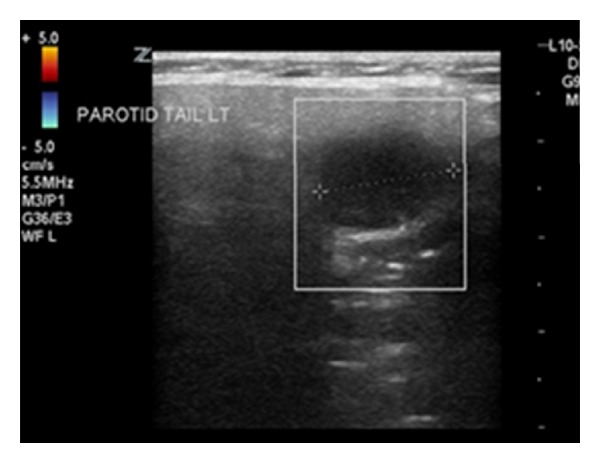
Ultrasound of parotid/neck region. Within the right parotid a 15 mm hypoechoic lesion was seen. Initial differentials: salivary gland tumour and abnormal possibly necrotic intraparotid node.

The patient underwent an urgent FDGPET and fine needle aspirate (FNA) ultrasound. The FNA ultrasound together with cytopathology (positive for CAM5.2 and CK20) was suggestive of MCC in the parotid (Figure 1). Immunocytochemistry was performed for confirmation.

The results of the investigations were discussed at both skin and head and neck multidisciplinary team (MDT) meeting with colleagues, and it was decided that the most appropriate management was a subtotal parotidectomy left side with ipsilateral selective neck dissection levels I to III.

## 4. Differential Diagnosis

The following is a differential diagnosis for MCC, and the list is by no means exhaustive [9]:

- basal cell carcinoma,
- small cell melanoma,
- lymphoma,
- blue round cell tumours,
- metastatic small lung carcinoma.

## 5. Treatment

An extra oral incision from the left preauricular region to the left neck was made and raised. Anterior, superior, and posterior flaps were developed in aid to carry out a selective neck dissection I–III with identification and preservation of the following anatomical structures:

- internal jugular vein,
- carotid artery,
- hypoglossal nerve,
- lingual nerve,
- greater auricular nerve,
- ansa cervicalis.

**Figure 2:**
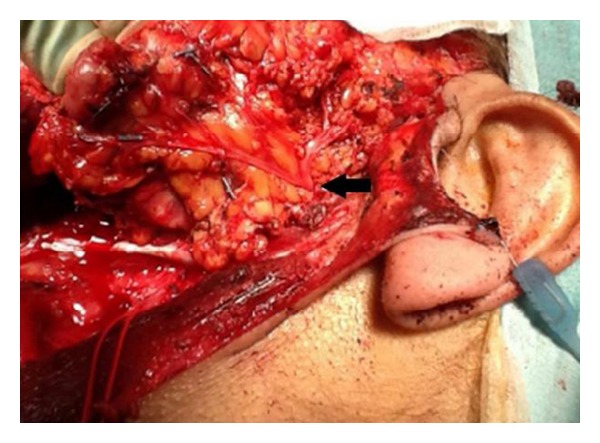
Careful dissection revealing the branches of the facial nerve which were preserved. The facial nerve passes through the parotid gland after emerging from the stylomastoid foramen. The arrow points to the point of division into five branches: temporal, zygomatic, buccal, marginal mandibular, and cervical.

A subtotal parotidectomy was carried out. There was identification and preservation of the nearby structures through careful dissection. The facial nerve passes through the parotid after emerging from the stylomastoid foramen, for this reason a parotidectomy carries the risk of damage to the branches of the facial nerve, in particular the marginal mandibular branch. The marginal mandibular branch of the facial nerve provides motor innervations to the muscles of the lower lip and the chin. Damage to this nerve can leave the patient with a drooping lip and chin on that side (Figure 2).

**Figure 3:**
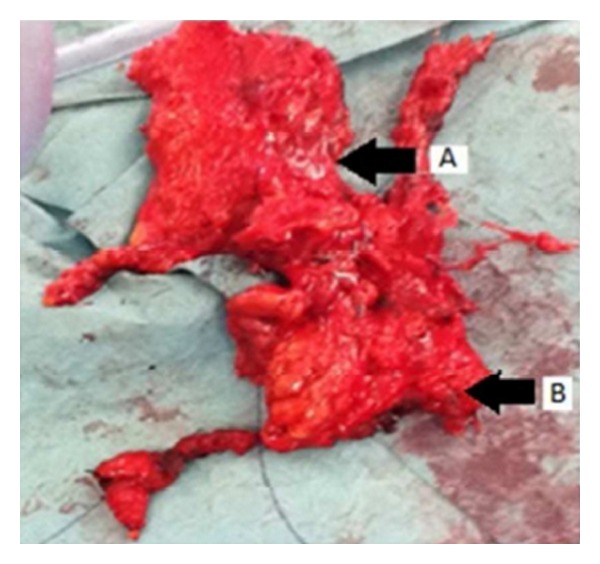
The specimen: the neck dissection attached to the parotid was removed as one entity. The neck dissection consisted of lymph nodes 1A, 1B, 2A, and 2B and level 3. A: body of parotid gland. B: level 2A nodes.

The specimen was removed with further deep harvest of salivary tissue on islands (Figure 3). Drains were placed and closure was carried out in layers.

## 6. Outcome and Followup

Histopathology revealed a well-sampled parotid gland with no metastatic tumour within salivary parenchyma. One intraparotid lymph node was almost completely replaced by a poorly differentiated tumour (20 mm in diameter) in keeping with metastatic MCC. There was no extracapsular spread. A final diagnosis of metastatic MCC left parotid was made.

The facial nerve was spared and immediately after surgery the patient had good facial expression and no signs of neurological deficit. At a short-term follow-up appointment the patient had no shoulder weakness and only slight weakness of his lower lip. As this weakness was not present immediately after surgery it is likely this is a temporary paresis due to post-op oedema. The patient is under regular followup and at present free of disease.

## 7. Discussion

It is important to note that the management of MCC must be conducted on a patient by patient basis. The patients with this disease are often elderly with a number of comorbidities which may not make them medically fit for surgical procedures. Radiotherapy is often a palliative candidate in such groups. Chemotherapy (cisplatin, doxorubicin, vincristine, and etoposide) responses have been shown to be unfavourable in the management of this disease [10, 11].

One treatment option is to look at surgical resection followed by adjuvant radiotherapy. A multicentre randomised control trial reveals good local and regional control; however, the results could not be described as statistically significant [12]. The other option is surgical removal of disease with local lymphatic dissection. Sentinel lymph node biopsy (SLNB) has been indicated in many studies when looking at MCC [13]; however, SLNB is extremely difficult in the head and neck and there is the possibility of an under diagnosis. Therefore, the surgical team needs to make an evidence based decision to whether an elective neck dissection will be conducted or not. In this case a full appreciation of the epidemiology, prognosis, and aggressiveness of the disease was highlighted to the patient and surgical team. A decision was made to electively dissect the left neck, levels I–III. There is evidence supporting this surgical decision. Brissett et al. [14] display 2-year survival rates for a group of 22 patients who were treated for MCC by (a) wide local excision (WLE) only, (b) WLE and local lymphage drainage basin dissection, or (c) Mohs Surgery (chemosurgery). At a 2-year followup all patients in group (b) WLE plus lymphage dissection, were alive. 68% who had WLE were alive at 2 years and 33% who had Mohs surgery.

The benefit of adjuvant radiotherapy is not to be undermined. Following surgical resection, radiotherapy can be used to irradiate the region, including localised regions of lymph nodes. This can be extremely important if frozen section analysis or histopathology postsurgery reveals margins which are still positive for disease.

## 8. Conclusion

As many lesions of MCC are on sun exposed areas of the skin, it is often dermatological colleagues who make the initial investigations and diagnosis before referring to surgical teams. This case report describes the surgical management of a recurrent MCC together with a selective neck dissection. Appreciation of the surgical pathway is important to give the patient and clinician a realistic depiction of the patient journey prior to referral.

## 9. Learning Points

1. The most published treatment management option for patients with head and neck MCC is surgical resection with adjuvant radiotherapy. The clinician should also be aware of the benefits of wide local excision with elective neck dissection in this rare and aggressive disease.
2. Taking an evidence based approach when planning patient management is necessary.
3. Exploring and critically appraising all treatment options with colleagues as well as the patient are necessary to ensure an informed decision is made, especially in rare cases where the chance of reoccurrence is high.
